# Taking care of our future doctors: a service evaluation of a medical student mental health service

**DOI:** 10.1186/s12909-020-02075-8

**Published:** 2020-05-29

**Authors:** Rebecca Jacob, Tsz-yan Li, Zoe Martin, Amanda Burren, Peter Watson, Rhian Kant, Richard Davies, Diana F. Wood

**Affiliations:** 1 Dept of Liaison Psychiatry, Cambridgeshire and Peterborough NHS Foundation Trust, Cambridge, UK; 2 Dept of Liaison Psychiatry, Addenbrookes Hospital, Hills Road, Cambridge, CB2 0QQ UK; 3 MRC Cognition & Brain Sciences Unit, University of Cambridge, Cambridge, UK; 4 School of Clinical Medicine, University of Cambridge, Cambridge, UK

**Keywords:** Higher education, Medical Student, Mental disorders, Fitness to practice, Service evaluation

## Abstract

**Background:**

Studies suggest medical students experience high levels of mental distress during training but are less likely, than other students, to access care due to stigma and concerns regarding career progression. In response, The School of Clinical Medicine, University of Cambridge supported the development of the ‘Clinical Student Mental Health Service’ to provide specialist input for this vulnerable group. This study evaluates the efficiency and effectiveness of this service.

**Methods:**

Using mixed-methods, cross-sectional analysis of validated psychiatric rating scales and qualitative feedback, 89 responses were analysed from 143 clinical students referred, between 2015 and 2019. The care pathway included initial review by a psychiatrist, who triaged students to psychologists delivering therapies including: Cognitive Behavioural Therapy, Interpersonal Therapy, Eye Movement Desensitization Reprocessing Therapy or Cognitive Analytic Therapy.

Efficiency was assessed by waiting times for psychiatry and psychology interventions, and number of sessions. Academic outcomes included school intermission and graduation. Clinical effectiveness was analysed by measuring global distress, depression, anxiety, functioning and suicidal risk. Pre/post intervention changes were captured using t-test and McNemar test with thematic analysis of qualitative feedback.

**Results:**

Referral rates increased from 3.93% (22/560) in 2015 to 6.74% (45/668) in 2018. Median waiting times for initial psychiatric assessment and start of therapy was 26 and 33 days, respectively. All graduating students moved on to work as junior doctors.

Levels of distress, (t = 7.73, p < 0.001, df = 31), depression (t = 7.26, p < 0.001, df = 34) anxiety (Z = − 4.63, p < 0.001) and suicide risk (Z = − 3.89, p < 0.001) were significantly reduced. Participant’s functioning was significantly improved (p < 0.001, 99.5% CI 4.55 to 14.62). Feedback indicated high satisfaction with the rapid access and flexibility of the service and the team clinicians.

**Conclusions:**

A significant proportion of medical students attending the service scored highly on validated rating scales measuring emotional distress, suicidality and mental illness. Reassuringly they benefitted from timely specialist mental health input, showing improvements in mental well-being and improved functioning. The development and design of this service might serve as an exemplar for medical schools developing similar support for their students.

## Background

Mental and emotional difficulties in Higher Education students are a growing public health concern, both within the UK and indeed worldwide [1, 2]. Whilst this phenomenon encompasses all academic disciplines, studies suggest the prevalence may be higher amongst medical students [3] particularly with respect to anxiety (25.7%), burnout (49.6%) and stress (31.2%) [4–6]. A meta-analysis of 183 studies across 43 countries showed that the prevalence of depression or depressive symptoms among medical students was 27.2 and 11% of them reported suicidal ideation [7], which is consistent with other studies [4, 5, 8]. Worryingly a 2018 British Medical Association meeting reported that six UK medical students completed suicide in an 18-month period [9]. As medical students transition to hospital and community training, they may become more vulnerable to mental illness. Commonly reported stressors at this stage include relationship difficulties with supervising consultants, possible humiliation and bullying during placements, too much or too little responsibility at placement, exposure to patients’ distress, terminal illness and death, compromised sleep patterns and the pressure of developing a professional persona [10–14]. Some of these pressures are self-imposed, such as setting the bar too high to reach, self-doubt, and equating performance to identity [15].

Despite their emotional distress, a UK-based study commissioned by the General Medical Council (GMC) showed that medical students were unlikely to seek help [15]. Perceived stigma associated with psychiatric disorder remains common among clinical students, as are an emphasis on the invincibility of doctors, misunderstandings about Fitness to Practise (FTP) procedures, concerns regarding career progression and peer comparison [15–19]. The learning environment during hospital placements, with frequent relocation, is another obstacle to seeking support [16, 19]. Prevalence, risk factors of mental health problems and barriers to accessing help among medical students have been extensively studied, but greater knowledge is needed regarding effective interventions to help these students [20]. Whilst there is a growing literature base describing how medical schools are supporting students to develop resilience to manage the significant demands of the course [21], the GMC has also highlighted the importance of ensuring access to appropriate mental health services for student doctors [22].

A previous study at the School of Clinical Medicine (later referred to as ‘the School’), University of Cambridge found the prevalence of depression varied from 2.2 to 10.0% among medical students in their ‘clinical component’ (Year 4 to 6; later referred to as ‘clinical students’. These are medical students who have embarked on hospital or community training placements). It was therefore suggested that mechanisms to identify and support students should be in place [23]. Students at the University are encouraged to seek pastoral care through their College tutorial and welfare systems, University-wide services such as the Student Union, the University Counselling Service, the Disability Resource Centre and the Occupational Health Service. However, once they start their placements, they are often located away from these support systems. We also identified a gap in local specialist mental health services, due to significant pressures within the National Health Services (NHS) and high thresholds for referral. To address the unique needs of clinical students, the School and the Cambridgeshire and Peterborough NHS Foundation Trust (CPFT) collaborated to develop the Clinical Student Mental Health Service (CSMHS) in 2015, which aims to provide easy and rapid access to support from a consultant psychiatrist and clinical psychologists. This paper evaluates the efficiency, effectiveness and students’ subjective experience of the CSMHS.

## Methods

### The CSMHS

The CSMHS is funded by the School, using a small proportion of the undergraduate tariff from Health Education England applied to the University of Cambridge. It is a non-urgent service, offering psychiatric and clinical psychology input over two/three days, respectively, in a week.

The service is delivered at the Liaison Psychiatry Department of Addenbrooke’s and Fulbourn Hospitals Cambridge. Students on regional placements are supported financially by the School for their travel and are not required to specify the nature of the appointment to placement supervisors.

**Fig. 1:**
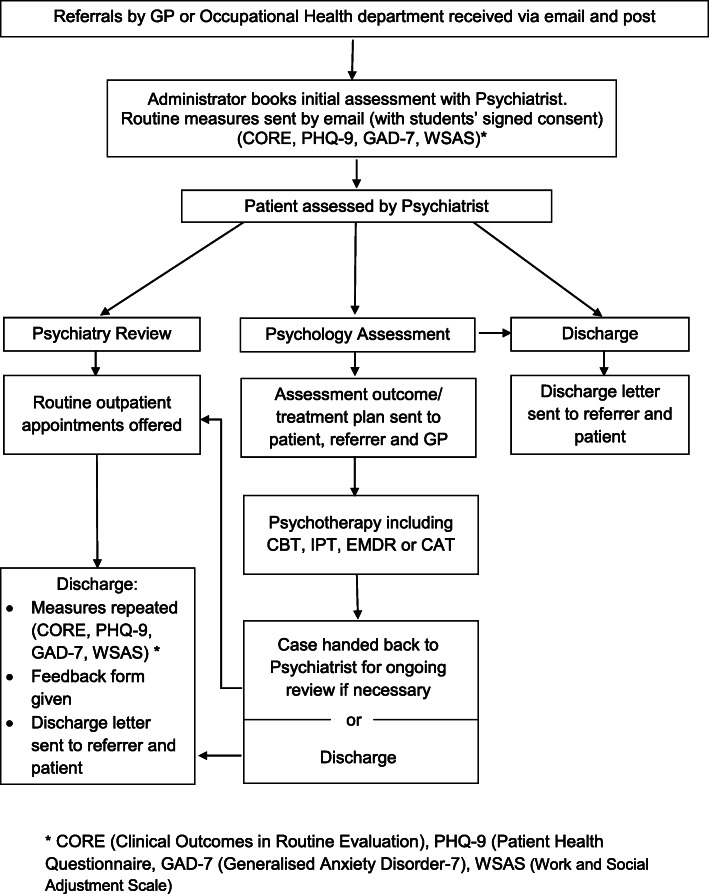
Clinical Student Mental Health Service (CSMHS) Process Map

The referral process of the CSMHS is illustrated in Fig. 1. Students are made aware of pastoral and welfare support systems when they first enter the School. Leaflets describing the CSMHS are also made available. Initially it was designed as a tertiary level service with referral by their University Occupational Health (OH) department. However, students regarded this route as somewhat convoluted, so direct primary care referrals are now accepted. The School does not refer directly, nor are they included in any way in the treatment process, ensuring confidentiality is maintained.

Following an initial psychiatric diagnostic assessment, a management plan is made jointly by the psychiatrist and student. If indicated, a referral will be made either to the CSMHS psychology service for therapy or other appropriate secondary NHS services. Decisions regarding who to refer for psychology are based on student choice and evidence-based guidance for psychological treatment of mental health conditions. Other considerations include presenting suicidal risk and the ability to attend and engage in a course of therapy sessions. If further input is not necessary, the student will be discharged to primary care. Psychiatry review clinics are offered when necessary or requested.

Students referred for CSMHS psychology service will be assessed by a clinical psychologist who develops collaboratively with the student, an evidence based tailor-made plan. The following therapies are available: Cognitive-Behavioural Therapy (CBT), Interpersonal Therapy, Eye Movement Desensitization and Reprocessing, or Cognitive Analytic Therapy. For therapies that are not available within the CSMHS, routine NHS referrals are also available. The length of treatment depends on an individual students’ need.

To calibrate the change in their mental health conditions and their experience of treatment, students complete the Clinical Outcomes in Routine Evaluation (CORE), the Generalized Anxiety Disorder-7 Scale (GAD-7), the Patient Health Questionnaire-9 (PHQ-9), and the Work and Social Adjustment Scale (WSAS) before and after treatment. After treatment, they also complete an anonymised qualitative feedback form.

Neither the psychiatrist nor the clinical psychologists are involved in assessing or tutoring at the Clinical School or in FTP procedures, to avoid conflicts of interest.

### Service evaluation

This service evaluation was registered and approved by the Quality Assurance and Clinical Effectiveness Unit, CPFT and funded by the National Institute of Health Research Applied Research Collaborations Easts of England (NIHR ARC EOE). Based on the Medical Research Council and NHS Health Research Authority guidelines neither separate ethics approval nor consent were required for evaluation of the anonymised dataset. The evaluation assessed waiting times, treatment delivery (intensity, treatment completion and dropout rate), academic outcomes, clinical progress and quantitative and qualitative feedback from the students.

Waiting times were defined as the time lag between initial referral and first psychiatric or psychology assessment, excluding those who delayed assessment due to declining an earlier appointment offered (for example, when students were away from University on placement).

Treatment duration was the total number of psychiatric review appointments and therapy appointments attended by students who completed treatment. Treatment completion was calculated as the percentage of students who had completed the intervention as a proportion of the total number of students who were offered a treatment.

Academic outcomes were assessed by years of intermission from their study, and whether they had successfully moved on to their Foundation Year.

Clinical outcomes in terms of global distress, anxiety, depression and perceived functioning were assessed respectively by CORE, GAD-7, PHQ-9 and WSAS that students completed before (T1) and after treatment (T2). Students’ subjective experience of the service was captured by anonymised qualitative feedback forms.

### Outcome measures

The CORE is a 34-item self-report rating scale with good reliability and convergent validity [24]. It measures the global distress of an individual and consists of four subscales; well-being (4 items), problems/symptoms (12 items), life functioning (12 items) and risk (6 items), rated from 0 (Not at all) to 4 (Most or all the time). Suicide risk is measured by the risk subscale. Mean scores are computed for all items and subscales with higher mean scores indicate higher level of difficulty. Validated, gender-specific cut-off scores differentiate those who fall into the clinical range from those who do not.

The GAD-7 is used to measure the severity of generalised anxiety disorder symptoms [25]. It is a validated and widely used self-report scale consisting of seven primary anxiety symptoms. Respondents are asked to rate symptoms in the past 2 weeks from 0 (Not at all) to 3 (Nearly every day). The clinical cut-off is five with higher scores indicating greater levels of anxiety.

The PHQ-9 is a well-validated 9-item self-report rating scale for depression [26]. The clinical cut-off is five with higher scores indicating higher levels of depression.

The WSAS is a validated, 5-item self-report scale which measures perceived functioning across five dimensions; work, home management, social leisure activities, private leisure activities, and family and relationships [27]. Impairment in each dimension is rated from 0 (Not at all affected) to 8 (Very severely affected) with higher scores indicating greater perceived impairment. No validated cut-off is available.

Scores (of the above measures) before (T1) and after treatment (T2) were compared by paired-sample t-tests for those variables with a normally distributed score differences (i.e. T2-T1), or the Sign test (for assessing the consistency of change) and the Wilcoxon signed rank test (for assessing the magnitude of scores differences) for those without a normal distribution. The McNemar test was used to compare the change in the proportion of students falling in the clinical range (i.e. scoring above the clinical cut-offs) of anxiety and depression after treatment. SPSS version 25.0 was used for the analysis.

### Student involvement and feedback form

Students were actively involved in the development of the service, including designing the referral pathway. A student on their Student Selected Component (SSC) conducted focus groups to canvass students’ views. Issues relating to a gap in NHS service provision were highlighted. FTP and performance were identified as areas that needed to be kept separate from our service.

When discharged from the service, students completed anonymous feedback forms to indicate how likely they would be to recommend the service on a 5-point scale ranging from 1 (Extremely likely) to 5 (Extremely unlikely), whether it was easy to access support (Yes/No), whether they felt listened to, taken seriously and treated with dignity and respect, whether their views were considered when agreeing a treatment plan, whether the input had helped them to cope with course demand, and the overall rating of the service on a 5-point scale ranging from 1 (Very good) to 5 (Very poor). They also indicated if they would recommend the service to peers, what was helpful and any suggestions for improvement.

The Yes/No questions and the 5-point scales were treated as categorical data and ordinal data, respectively. Descriptive statistics were computed for these questions. Two independent raters, who were not involved in the delivery of the service, analysed the feedback, and identified common themes that occurred in the written text.

## Results

### Service coverage

Over the service evaluation period (January 2015 to February 2019), 143 referrals were received. Over this time the School was expanding their student numbers, however, after controlling for this, the number of referrals increased yearly. We received 22 referrals between 2015 and 2016 (3.93%, 22/560), 28 referrals between 2016 and 2017 (4.94%, 28/567) and 45 referrals between year 2017–2018 (6.74%, 45/668). Overall, the service supported 5.29% of all clinical students from years 2015–2018.

### Participants

Of those clinical students referred, 89 completed the routine measures (Male = 39 (43.82%); Females = 50 (56.18%); Mean age = 23.26; See Table 1**)**.

**Table 1:** Demographic characteristics of participant

Characteristics	*N*	%
Participants (Mean age = 23.26)	89	
Sex		
Male	39	43.82
Female	50	56.18
Ethnicity		
White	44	49.44
Black	3	3.37
Asian	25	28.09
Mixed	3	3.37
Not specified	14	15.73
Education		
Year 4–6	79	89.76
Graduate	6	6.74
MBPhD	3	3.41
Psychiatric diagnosis		
Adjustment disorder	29	32.58
Depression	29	32.58
Anxiety disorders	17	19.10
Obsessive-compulsive disorder	10	11.24
Personality disorders	8	8.99
Irritable Bowel Syndrome	5	5.62
Eating disorders	3	3.37
Others (e.g. Bipolar affective disorder, life-management difficulty, schizophrenia)	9	10.11
More than 1 disorder	21	23.60
Source of referral		
GP	49	55.06%
Occupational Health Department	40	44.94%

Seventy-two students (80.90%) had sought help from other mental health services (e.g. their GP, the University Counselling Service, other psychology service) before they were referred to us. Fifty-five students (61.8%) had no psychiatric history before they entered medical training.

Only 3/89 students were referred to the FTP committee by the Clinical School. None of these referrals were directly related to their mental health issues. Independent psychiatrists provided reports for the panel if required.

### Waiting time

The distribution of the waiting time from the day of referral to the first assessment was positively skewed. The median waiting time was 26 days, with the 25th and 75th percentiles of 12.25 days and 38.25 days, respectively. Similarly, the waiting time from psychology referral (made by the psychiatrist after the initial assessment) to the first psychology assessment was also positively skewed, with a median of 33 days, 25th percentile of 18.75 days and 75th percentile of 43.25 days.

### Treatment delivery

Both the number of psychiatry sessions (median = 1; ranged from 1 to 11) and the number of therapy sessions (median = 8; ranged from 1 to 26) showed a positively skewed distribution.

Sixty-one students (68.54%) were prescribed psychotropic medications by the psychiatrist, of which 60 of them (98.36%) were prescribed antidepressants, three (4.92%) had antipsychotics, three (4.92%) had mood stabilizers. Ten students (16.39%) had more than 1 type of medication.

Of the 49 students who were offered psychological therapy, 44 of them (89.80%) received CBT, and seven of them (14.29%) received other psychotherapies. Two students received two types of therapy within one episode of care (4.08%). At the end of the evaluation period, 30 students (61.22%) had completed their treatment, one student intermitted (2.04%) and 10 students (20.41%) were receiving ongoing treatment. Eight students did not complete their treatments (16.33%). Five of these eight students dropped out of therapy and three no longer had access to the service due to graduation.

### Academic outcomes

Within the evaluation period, 28 students in our sample graduated from the School. All of these students moved on to work as junior doctors within UK Foundation Schools.

The School reported that a total of 39 students intermitted (took a complete break from their studies) during the period of our evaluation. Almost half of these (16/39) were students within our sample, who took time off on mental health grounds. At the time of writing, all of these 16 students had been able to return to Cambridge and continue with their studies.

### Clinical outcomes

Table 2 presents the comparisons before and after treatment on clinical outcomes. Statistically significant improvement (i.e. score reductions) was observed across all measures. Students appeared to become less distressed (*p* < 0.001), less depressed (*p* < 0.001), less anxious (sign test: *p* < 0.001; signed rank test: *p* < 0.001) and reported improved functioning (*p* < 0.001). Their suicidal risk was also reduced (sign test: *p* < 0.001; signed rank test: *p* < 0.001).

**Table 2:** Clinical outcomes

			Before treatment			After treatment								
Outcome measures	Max score	*N*	Mean	*SD*	Median	Mean	*SD*	Median	Mean difference (99.5% CI)	*SD*	Cohen’s *d*	*t*	*df*	*Z*
CORE	4	32	1.72	0.64		0.78	0.53		0.94 (0.57 to 1.30)	0.68	1.37	7.75**	31	
CORE(−risk)	4	32	1.99	0.72		0.92	0.61		1.07 (0.65 to 1.48)	0.78	1.37	7.74**	31	
Well-being	4	32	2.16	0.93		0.99	0.80		1.17 (0.62 to 1.71)	1.02	1.15	6.49**	31	
Problems	4	32	2.24	0.73		1.04	0.70		1.20 (0.74 to 1.66)	0.86	1.40	7.90**	31	
Functioning	4	32	1.68	0.78		0.78	0.53		0.89 (0.50 to 1.29)	0.73	1.22	6.90**	31	
Risk	4	32	/	/	0.25	/	/	0.00	/	/	−1.06 ^a^ (−1.58 to −0.53)	/	/	−3.75** ^a^
											−1.11 ^b^ (−1.64 to −0.58)			−3.89** ^b^
PHQ-9	27	35	13.20	6.91		5.29	4.57		7.91 (4.95 to 10.87)	5.83	1.36	8.03**	34	
GAD-7	21	35	/	/	10.00	/	/	4.00	/	/	−1.49 ^a^ (−2.01 to −0.95)	/	/	−4.77** ^a^
											−1.33 ^b^ (−1.85 to −0.81)			−4.63** ^b^
WSAS	40	29	19.93	9.25		10.34	7.37		9.59 (4.55 to 14.62)	8.90	1.08	5.80**	28	

^a^Cohen’s *d* and Cohen’s *d* 95% CI (in brackets) of Sign test

^b^Cohen’s *d* and Cohen’s *d* 95% CI (in brackets) of Wilcoxon Signed Rank test

**p < 0.001

After treatment, a significant portion of the students moved to score below the clinical cut-offs in terms of distress (62.5%, Exact *p* < 0.001), depression (48.6%, Exact *p* < 0.001) and anxiety (45.7%, Exact *p* = 0.001; **See** Table 3).

**Table 3:** Comparison of the proportion of students scoring below or above clinical cut-offs after treatment

Outcome measures	*N*	Became below cut-off	Became above cut-off	Remained below cut-off	Remained above	McNemar Exact *p* (2-tailed)
CORE	32	20 (62.5%)	0 (0%)	6 (18.8%)	6 (18.8%)	< 0.001
CORE(−risk)	32	21 (65.6%)	0 (0%)	6 (18.8%)	5 (15.6%)	< 0.001
Well-being	32	20 (62.5%)	2 (6.3%)	7 (21.9%)	3 (9.4%)	< 0.001
Problems	32	20 (62.5%)	0 (0%)	5 (15.6%)	7 (21.9%)	< 0.001
Functioning	32	18 (56.3%)	0 (0%)	8 (25.0%)	6 (18.8%)	< 0.001
Risk	32	10 (31.3%)	0 (0%)	19 (59.4%)	3 (9.4%)	0.002
PHQ-9	35	17 (48.6%)	0 (0%)	4 (11.4%)	14 (40.0%)	< 0.001
GAD-7	35	16 (45.7%)	2 (5.7%)	2 (5.7%)	15 (42.9%)	0.001

### Student feedback

Fifty out of 60 (83.33%) respondents rated they were extremely likely to recommend our service to their peers. All respondents rated that they felt they were treated with dignity and respect by the staff. Fifty-nine out of 60 respondents (98.33%) felt that their views were considered when approving a treatment plan. Forty-eight out of 56 respondents (85.71%) agreed that the input received from the service helped them cope better with course demands. Twenty-nine out of 30 respondents (96.67%) stated that it was easy to access support from the service and all respondents agreed that the team listened to and treated their concerns seriously. The service was given an overall rating of ‘Very Good’ by 50 out of 59 respondents (84.75%). Detailed results of thematic analysis on 53 respondents who provided written feedback is summarised in Table 4.

**Table 4:** Summary of thematic analysis categories from students’ responses on the Feedback Form (*N* = 53)

Category	Sub-category and Quotes	Number of students (*n*)	Percentage (%)
**Clinical students’ satisfaction with the service**	**Helpful and supportive** *“Brilliant service...Helped me a lot****.****”* “*… and extremely helpful”* *“It’s very comforting knowing that this service exists.”* *“Supportive & non-judgmental”*	18	33.96
	**Improvement and recovery** *“It made all the difference on my recovery”* *“I saw significant improvement with my issues/finally start to see some progress.”*	4	7.55
	**Contrast to other services** *“Listened & gave me more suitable treatment than that through IAPT”* *“Much quicker response than standard NHS process.”* *“Understands the difficulty faced by clinical students better than other services”*	10	18.87
	**Increased service awareness** *“Awareness could be better, I was only made aware by staff months after taking time off.”* *“More advertising to clinical school about this service”*	9	16.98
**Tailor-made treatments**	**Specialised to medical students** *“I feel like you really understand how it can be difficult for us to fit in sessions because of the course and you try to accommodate that.”* *“Understanding around demands of being medical student (tailored support)”* *“This service is tailored for clinical students, they are familiar with the particular problems and challenge”*	13	24.53
	**Person centred-care** *“My concerns and questions are considered and acted upon.”* *“Personal service”* *“I had input into the treatment plan and the direction it should go in.”* *“feel like will support me in what I want to achieve”*	18	33.96
	**Suitable medications or therapy** *“Psychiatric input finding right medication for me”* *“Psychological therapy was really helpful.”* *“CBT (has) given me the tools to manage my condition more effectively on a day to day basis/long term... thank you for helping me to learn how to manage things more effectively”* *“The CBT was extremely useful.”*	11	20.75
**Positive perception of clinicians**	**Compassionate, kind and respectful manner** *“Therapists have respectful and caring manner”* *“Amazing therapist and amazing team.”* *“Incredibly understanding and encouraging.”* *“She was kind and understanding of what I was going through”*	36	67.92
	**Feel comfortable, understood and listened to** *“Made me feel comfortable talking about things that I felt were to(o) difficult to talk about”* “She gave me a lot of time to express what I was feeling and my frustration for not finding anyone to help …. I felt comfortable disclosing personal or embarrassing thoughts.” “Feels like I’m listened to.”	18	33.96
**Service barriers and facilitators**	**Ease of accessibility** *“The accessibility was superior to that of the normal mental health referral routes”* *“Tt has been very easy to make appointments when I’ve needed them”* *“It’s easy to access”*	16	30.19
	**Rapid response** *“Rapid, Personal service”* *“Very fast response and a great help”* *“Quick response and appointment times”*	16	30.19
	**Flexibility** *“Length and frequency of appointments are very good”* *“Flexible appointments- always happy to see me when I needed it.”* *“Very supportive of my needs and flexible regarding placements”*	11	20.75
	**Confidentiality** *“Having somewhere else to wait for appointment in the liaison psych block at Addenbrookes. I often see other students on their placement whilst I am waiting for my appointment which does not feel very confidential.”* *“Find a way to see staff members without other students seeing you are waiting for an appointment”*	3	5.66

## Discussion

It is encouraging that many medical schools are developing mental and emotional support systems for their students [3, 22]. These include providing pastoral support from university tutors, developing university counselling services and adopting a third-party occupational health approach [16] There are similar pastoral support services developed across the UK both for medical students and doctors although these, in the main, provide counselling support [28]. The current study is one of the few evaluations of a specialist mental health service dedicated to clinical students. Karp and Levine published an evaluation of their service in the Pittsburgh School of Medicine which provides care for all medical students [3]. However, their treatment pathway is slightly different to the CSMHS; the majority of  their students are initially assessed by psychologists, and those with severe mental illness or who require medication management are subsequently referred to a psychiatrist.

Research has suggested that up to 27% of medical students suffer from depression/depressive symptoms [7]. Although referral rates increased yearly, we were surprised that only approximately 5% of clinical students, over the study period, were referred to this service. This may imply that other students, or their general practitioners, felt their symptoms were not severe enough to warrant specialist care, there was a lack of awareness of the existence of the service or, possibly, as previous research highlights, that stigma or other barriers to seeking help still exist [17–19]. Of the sample studied, 61.8% of the 89 students developed mental health issues after starting the medical course. Eighty percent had also accessed other mental health support**,** prior to attending the CSMHS. This concurs with the literature indicating that the nature of medical education in itself, may be a factor in students developing mental disorders [29]. Studies also report that emotional factors play a role in significant levels of dropout from medical education [30]. Reassuringly students in our sample despite, on occasion, intermitting from studies, were able to advance in their medical career, and all our graduating students had moved on to Foundation Year Training.

Overall, our service was received positively by the students. Specifically, they appreciated that a specialist mental health service for clinical students was made available and indicated they were extremely likely to recommend our service to other students. In terms of service delivery, feedback highlighted a strength of our service, compared to other medical schools and local NHS services, was the fact that students accessed the service easily and received prompt support with short waiting times (usually less than a month) with approximately a further 4 weeks to see a clinical psychologist for therapy. Student feedback also highlighted an appreciation for flexibility regarding the number of sessions provided. A limitation of this service was the fact it was located in an NHS setting which resulted in students being concerned about confidentiality. Therefore, a longer-term aim for the CSMHS is to situate care outside an NHS setting.

Our service outcome analysis suggested significant improvements in terms of levels of distress, depression, anxiety, suicidal risk and perceived functioning. A large proportion of the students scored below clinical cut-offs after treatment, indicating the service was associated with recovery. Indeed, students’ feedback reflected this improvement, which helped them to cope with the demands of the course and in other areas such as interpersonal relationships. The psychiatrist and the clinical psychologists were described as compassionate and understanding.

Contrary to students’ concern about FTP procedures in the context of mental health disorders, reassuringly only three (2.10%) of our referred students were involved in the procedure and these were for reasons not specifically related to their mental illness.

### Strengths and weaknesses of the study

A strength of this service evaluation is that both quantitative and qualitative data were analysed. The written feedback from the students offered additional evidence to the positive changes observed from the outcome measures, and this also allowed us to understand roughly the context of such changes. Using independent raters, who were not involved in the delivery of care, to conduct the thematic analysis, hopefully reduced bias.

Previous research has shown that students may be wary of support services that take place on campus (for example their College Tutor) and have concerns about confidentiality or even compromising academic progress, when disclosing mental health problems to their academic supervisors [17]. A possible strength of this service is that it is provided by health care professionals who do not play a role in tutoring or assessing the students.

There are several limitations, some associated with retrospective analyses in general. Specific to this study, our rating scales might have failed to capture improvement in disorders other than the ones measured, because the measures were specific and limited to symptoms of distress, depression, generalised anxiety and perceived functioning. Moreover, comparisons made before and after treatment were based only on the responses of the students who had completed the rating scales, which may create a bias in our analysis. Nevertheless, every effort was made to administer the rating scales to all students attending the service to minimize bias. A further limitation was that it was not ethically desirable and feasible to have a control group to compare those who had received the intervention and those who had not. Students were referred to us for help in managing their mental health so that they could cope better with challenges during clinical studies. Our goal therefore was to deliver prompt and effective treatment.

This service evaluation is the first step in examining the short-term change in the well-being of medical students following specialist mental health treatment. Future studies may focus on the longer-term change in career resilience in terms of the skills (such as internal locus of control, flexibility, capacity for emotional expression and problem-solving skills) that are highly likely to be strengthened through these interventions. It will also be helpful to evaluate whether the therapeutic gain is sustained in terms of how many of these students have achieved their aspiration to work as a fully qualified doctor and most importantly, how they are functioning in their role.

## Conclusions

Medical students may not seek help for mental health conditions due to stigma, a fear of compromising career progression and the pressures of medical training. Our work has shown clearly that a significant proportion of students score highly on validated rating scales measuring emotional distress, suicidality and mental illness.

Reassuringly, the provision of a psychiatric-led assessment service, which provides evidence-based psychological treatment, appears to provide an efficient means of supporting medical students who are struggling with their mental health. Initial data using repeated assessments clearly indicates that this service is perceived to be both welcome and, importantly, effective for this vulnerable population.

In a wider context, the development and design of the CSMHS might serve as a possible service model for other medical schools developing mental health services for their students. It is our view that prompt interventions for mental disorders is justified in this vulnerable group, and that a dedicated medical student mental health service will hopefully improve students longer term mental well-being, resilience and career trajectory in their chosen medical specialities.

## Data Availability

The datasets used and/or analysed during the current study are available in an anonymised format from the corresponding author on reasonable request.

## Abbreviations

GMC: General Medical Council
FTP: Fitness to Practise
‘the School’: School of Clinical Medicine, University of Cambridge
‘clinical students’: Medical students who have started hospital training
NHS: National Health Services
CPFT: Cambridgeshire and Peterborough NHS Foundation Trust
CSMHS: Clinical Student Mental Health Service
OH: University Occupational Health
Clinical Psychologist: Psychologists with Postgraduate / Doctoral level qualification in psychological assessment and therapy of diagnosed mental disorders
NIHR ARC EoE: National Institute of Health Research, Applied Research Collaboration East of England
CBT: Cognitive-Behavioural Therapy
CORE: Clinical Outcomes in Routine Evaluation
GAD-7: Generalized Anxiety Disorder Scale
PHQ-9: Patient Health Questionnaire-9
WSAS: Work and Social Adjustment Scale
